# Supercritical Extraction of Red Propolis: Operational Conditions and Chemical Characterization

**DOI:** 10.3390/molecules25204816

**Published:** 2020-10-20

**Authors:** João Henrique de Oliveira Reis, Bruna Aparecida Souza Machado, Gabriele de Abreu Barreto, Jeancarlo Pereira dos Anjos, Larissa Moraes dos Santos Fonseca, Alex Alisson Bandeira Santos, Fernando Luiz Pellegrini Pessoa, Janice Izabel Druzian

**Affiliations:** 1 School of Pharmacy, Federal University of Bahia (UFBA), Barão de Jeremoabo Street, 147, Salvador 40110-100, Bahia, Brazil; jhonyba47@hotmail.com (J.H.d.O.R.); janicedruzian@hotmail.com (J.I.D.); 2 SENAI Institute of Innovation (ISI) in Advanced Health Systems (CIMATEC ISI SAS), University Center SENAI CIMATEC, National Service of Industrial Learning—SENAI, Orlando Gomes Avenue, 1845, Salvador 41650-010, Bahia, Brazil; gabriele.barreto@fieb.org.br (G.d.A.B.); jeancarlo.anjos@fieb.org.br (J.P.d.A.); larissa.fonseca@fbter.org.br (L.M.d.S.F.); alex.santos@fbter.org.br (A.A.B.S.); fernando.pessoa@fieb.org.br (F.L.P.P.)

**Keywords:** red propolis, supercritical fluids, carbon dioxide, ethanol, phenolic compounds, antioxidants

## Abstract

The objective of this study was to determine the best operational conditions for obtaining red propolis extract with high antioxidant potential through supercritical fluid extraction (SFE) technology, using carbon dioxide (CO_2_) as the supercritical fluid and ethanol as the cosolvent. The following parameters were studied: overall extraction curve, S/F (mass of CO_2_/mass of sample), cosolvent percentage (0, 1, 2 and 4%) and global yield isotherms as a function of different pressures (250, 350 and 450 bar) and temperatures (31.7, 40 and 50 °C). Within the investigated parameters, the best conditions found were an S/F of 131 and the use of ethanol at the highest concentration (4% *w*/*w*), which resulted in higher extract yields and higher content of antioxidant compounds. Formononetin, the main biomarker of red propolis, was the compound found at the highest amounts in the extracts. As expected, the temperature and pressure conditions also influenced the process yield, with 350 bar and 40 °C being the best conditions for obtaining bioactive compounds from a sample of red propolis. The novel results for red propolis found in this study show that it is possible to obtain extracts with high antioxidant potential using a clean technology under the defined conditions.

## 1. Introduction

Propolis is a natural compound defined as a complex resin matrix produced by bees from a mixture of exudates from different plants, wax and salivary secretions; it has major applications in the food, pharmaceutical and cosmetic industries [1,2]. Numerous studies have demonstrated its antioxidant [3,4], antimicrobial [5,6], anti-inflammatory [7,8] and antitumor [7,8] activities, among others [9,10,11,12]. These activities are attributed to the bioactive chemical compounds in propolis, such as phenolic acids, flavonoids, terpenes and sesquiterpenes [13,14,15]. Given this context, research has intensified on different extracts from natural matrices that have a wide variety of compounds with beneficial effects on health, such as propolis [16,17,18,19]. Among the various types of propolis, classified according to their physicochemical properties and geographical location, red propolis has gained prominence due to its composition and pharmacological properties [20,21,22]. Originating in Northeast Brazil, red propolis has biologically active flavonoids as its major compounds and may also contain cinnamic acid derivatives, esters and some terpenes [14,22,23]. Flavonoids are the most common and most varied group among the phenolic compounds of red propolis, play a role in different physiological processes and perform different functions, including beehive protection [13,14,22].

In natural matrices, the extraction method plays a critical role in the types of components obtained and extraction yield, and methods that involve the use of organic solvents such as ethanol, ethyl ether, methanol and chloroform are the most commonly employed. Much of the work involving propolis is performed using the conventional extraction process (hydroalcoholic) to obtain its extracts [20,24,25]. However, despite being commonly employed, conventional extraction has some disadvantages, mainly because it is a slow process that presents solvent residues in the final product. Due to increasingly restrictive environmental regulations and new trends related to “green chemistry principles”, extraction with supercritical fluids (SFE) has gained special interest among extraction techniques and is also considered a differentiated route for the extraction of compounds/substances from natural matrices [26]. This technology has been disseminated in multiple industrial areas due to its unique advantages and to the characteristics of supercritical fluids, such as diffusivity and viscosity close to those of a gas, a density similar to that of a liquid, low extraction temperatures, selective extraction, simplicity and recovery of the solvent-free product, and generating products with high added value at the end of the process [21,27]. In addition, SFE presents good yields and preserves the physico-chemical characteristics of the components to be extracted, presenting numerous advantages over conventional extractions, mainly considering the greater selectivity of the process [27,28,29]. Important aspects that should be considered in SFE are the choice of operational conditions in the extraction process for each studied matrix [27,29,30], i.e., the use of optimized values for the different conditions (pressure, temperature, solvent flow rate and volume, cosolvent type and concentration, among others) can significantly improve the yield and recovery of the compound or class of compounds of interest [31,32].

Despite being considered a promising alternative, few studies have used SFE to obtain propolis extracts, especially with regard to the evaluation of process conditions [20,33,34]. A previous study by our group determined the process conditions for the extraction of artepillin C and *p*-coumaric acid from green propolis [33]. Fachri et al. [34] also demonstrated the influence of pressure, temperature and CO_2_ mass flow rate on the extraction yield of Indonesian propolis, while Biscaia et al. [35] performed a comparative study of different extraction methods for green propolis. In addition, different studies and patents have shown that SFE has a high potential to improve the quality of the propolis extract and to increase the aggregate value of this matrix [36,37,38,39,40]. This novel study is an investigation of the best process conditions for obtaining compounds of interest from red propolis by SFE. It is important to highlight that red propolis possesses distinct biological properties and chemical compositions from the other types of propolis. For example, red propolis is mainly composed of active flavonoids and may also contain cinnamic acid derivatives, esters and some terpenes [23], while green propolis is well characterized by the presence of prenylated phenylpropanoids (e.g., artepillin C) and caffeoylquinic acids [41]. This characterizes each propolis as two different samples and, therefore, each one should present a different extraction profile and should be analyzed differently, once the molecular structure of the compound contributes to the difference in extraction behavior in SFE [42].

Given this context, the objective of this study was to determine the best extraction conditions for obtaining red propolis extract with high antioxidant potential using carbon dioxide (CO_2_) as a supercritical fluid and ethanol as a cosolvent. For this purpose, the overall extraction yield (X_0_) and extraction kinetics, determined from the overall extraction curves (OECs), were obtained, different cosolvent percentages were investigated and the global yield isotherms (GYIs) were obtained for total phenolic compounds, antioxidant activity and for the main compounds of interest present in red propolis (formononetin, naringenin and kaempferol).

## 2. Results and Discussion

### 2.1. Extraction Kinetic Curve and S/F

The first study step involved the determination of the supercritical extraction kinetic curve of propolis using CO_2_ as a solvent for the purpose of determining the amount of CO_2_ required for the diffusion period of the process to be reached and for the process time to be estimated. The literature shows that extraction kinetic curves can be divided into three phases. The first is related to the constant extraction rate (CER) phase, when the solute is easily accessible on the surface of matrix particles; the second represents the falling extraction rate (FER) phase, when the mass transfer rate decreases rapidly as a result of the decrease in the effective mass transfer area; and last, the diffusional period (DP), characterized by the absence of an easily accessible solute in the third phase [43]. Thus, and as evidenced in different studies, obtaining the diffusional period is of great importance to ensure that the global yield tests present results close to the real exhaustion of the extraction bed [44].

To obtain the diffusional period in the present study, the bed was filled with 7.5 g of red propolis sample, and the extraction was performed under temperature and pressure conditions that were milder than those studied (40 °C and 100 bar, solvent density of 1.8204 kg/m^3^). A total of 13 experiments were obtained for each assay used. Table 1 shows the results obtained for each experiment in relation to the S/F ratio, the mass yield of extract, the accumulated yield percentage and the mass of extract (g), and the yield for the content of total phenolic compounds and antioxidant activity. The total yield under the conditions used was 5.89% after a period of 5 h and 42 min of extraction using 0.81 m^3^ CO_2_. With the increase in the dynamic extraction time at constant pressure and temperature, an increase in the efficiency of the whole process was observed, and therefore, an increase in the yield of extracted compounds was expected [45].

In studies on the extraction of compounds from the herbs *Plantago major* and *Plantago lanceolata*, Mazzuti et al. [44] obtained fully developed kinetic curves, reaching the diffusional period at 240 min, using 15 g of crude extract from the samples defined for a pressure condition of 240 bar and a temperature of 50 °C. The same kinetic curve profile was found by Teixeira et al. [46] when analyzing the impact of temperature and pressure on SFE tests using 15 g of pracaxi seed oil (*Pentaclethra macroloba*), reaching the diffusional period at 240 min under the conditions of 40 °C and 250 bar.

**Figure 1:**
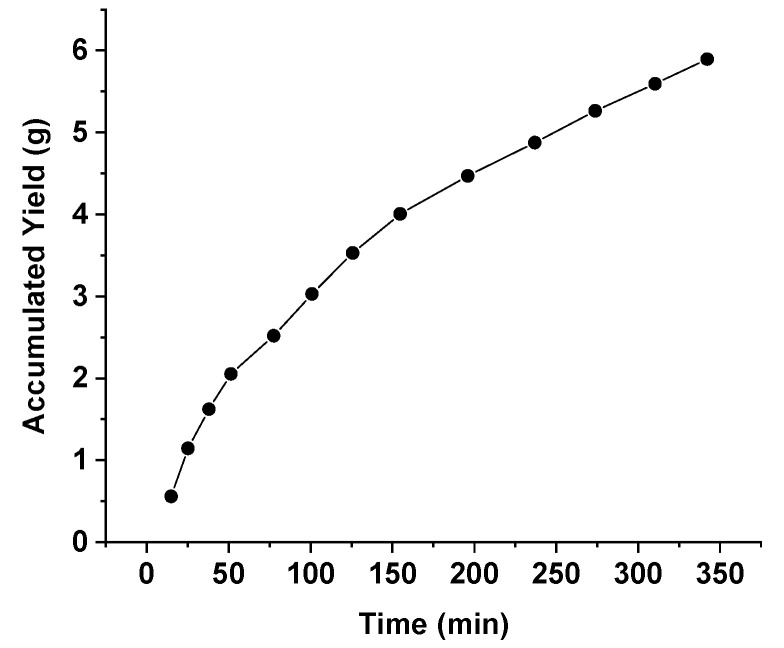
Kinetic curve obtained for the mean accumulated extract mass versus the total extraction time of the propolis sample.

Figure 1 shows the kinetic curve obtained for the mean accumulated extract mass versus the total extraction time of the propolis sample. According to different studies, extraction kinetics experimental data are fundamental for scaling up studies of the supercritical technology applied to obtain extracts of solid matrices [44,46,47]. Using the SFE technology in natural matrices, different studies have shown that the kinetic curve is not a linear function of time, and its shape is an indicator that different mechanisms control the mass transfer in the different extraction stages [44,48,49]. In the curve obtained for red propolis (Figure 1), a classical pattern that occurs in SFE was observed, that is, the presence of a period of constant extraction rate in the first hours. This is related to the extraction of substrates that are easily accessible to supercritical CO_2_ (process solvent). Subsequently, there is a progressive reduction in the extraction rate over time, which represents the extraction of substrates that are difficult to access by the supercritical solvent [42,50].

**Figure 2:**
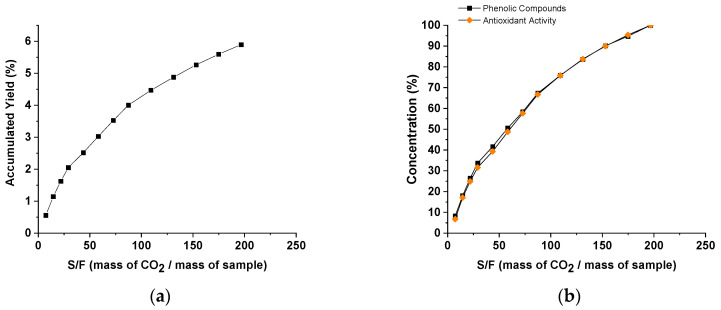
Results obtained for the overall extraction curves for (**a**) yield in mass (g) of accumulated extract versus S/F and (**b**) yield of phenolic compounds (%) and antioxidant activity (%) versus S/F (mass of CO_2_/mass of sample) (cumulative values).

The extraction kinetics of red propolis using CO_2_ as a supercritical fluid are also represented by the overall extraction curves (Figure 2). Figure 2a shows the yield of accumulated extract (accumulated mass) during the process as a function of S/F, while Figure 2b shows the global yield of phenolic compounds and antioxidant activity versus S/F considering the total extraction time.

Based on the results presented for study step 1 and after analyzing the extraction results and behavior using CO_2_ as a supercritical fluid under the extraction conditions defined, an S/F of 131 was determined because more than 80% of the phenolic compounds were extracted, representing 63% of the global yield in mass of the extraction. At this point of the relationship between the solvent mass and sample mass, an accumulated yield of 4.88% (63% of the total mass extracted) was obtained, where a total CO_2_ volume of 0.540 m^3^ with an approximate extraction time of 240 min was obtained. Albuquerque et al. [43] obtained extracts rich in tocotrienols and defatted bixin-rich seeds from annatto and determined an S/F ratio of 35. Similar behavior was observed by Machado et al. (2012) [33], who determined the extraction kinetics curve for green propolis with a total extraction time of 150 min and an S/F ratio of 110. In the study by Krakowska-Sieprawska et al. [26], the highest yields were obtained at 60 and 120 min of the process and reached 6.42% for extracts from yerba mate and 9.60% for extracts from yellow lupine, respectively.

The low mass yield of propolis extract by SFE has been demonstrated in other studies, which may be related to the fact that propolis is not very soluble in supercritical CO_2_ but can be much more soluble in a mixture containing CO_2_ and ethanol [51,52,53]. SFE is considered a very efficient technique in terms of selectivity for the extraction of target compounds, as well as for the separation and fractionation of different classes of compounds; however, depending on the polarity of these compounds, it is necessary to add small amounts of a modifier or cosolvent to the system to improve process yield [54]. For this reason, the influence of ethanol as a cosolvent at different concentrations in obtaining red propolis extracts by SFE with high antioxidant capacity was also evaluated in this study.

### 2.2. Determination of the Cosolvent Concentration

Although CO_2_ is the most commonly used solvent in SFE, one of the main limitations of its use is the reduced capacity to dissolve polar molecules, as is the case for the compounds present in red propolis, even at high densities [55]. Phenolic compounds and flavonoids are the bioactive components of propolis of major importance from a biological significance standpoint due to their pharmacological activities and their applicability in different areas [56]. It is well documented that the total content of phenolic compounds present in propolis varies according to several factors, including the extraction method and conditions used [57]. Regarding red propolis, previously studies have reported the presence of more than 300 components, which are representatives of terpenes, flavonoids, aromatic acids and fatty acids [22]. Flavonoids, are the most common and widely distributed group of phenolics in red propolis, being the most active compound in this natural matrix, with formononetin as the most relevant chemical marker of this type of propolis [58].

Due to the hydrophilic properties of phenolic compounds, to increase the extraction capacity of these compounds using SFE, soluble polar solvent (e.g., methanol, ethanol, and water) can be added to supercritical CO_2_ to modify its properties during extraction. In the present study, the SFE conditions using ethanol as a cosolvent were also evaluated. Table 2 presents the results for the yields of phenolic compounds, flavonoids, antioxidant activity (IC_50_-DPPH●), and concentrations of the formononetin and kaempferol compounds using 1, 2 and 4% ethanol (cosolvent) in relation to the mass of CO_2_ (*w*/*w*) and without the presence of a cosolvent.

Based on these data, it was observed that the best extraction conditions to obtain a higher yield of the compounds of interest—formononetin and kaempferol—as well as the highest content of total phenolics and antioxidant activity (represented by the lowest IC_50_) were achieved when 4% cosolvent was used (highest concentration investigated in this study). The extraction yields of total phenolics from red propolis can be increased by up to 57% with the presence of ethanol in the system as a cosolvent to supercritical CO_2_. Furthermore, the antioxidant capacity of the extracts was increased by 70%, thus demonstrating that it is possible to increase the solubility of antioxidant compounds in supercritical CO_2_ by adding ethanol. This may be related to the increased polarity of the solvent together with the ability of ethanol to improve the extraction surface area in a natural solid matrix [59]. The expansion of the material, generated by the interactions between solids and solvents, and the affinity between the liquid solvent and the associative compounds in the raw material leads to greater miscibility of the gas in the sample, resulting in greater extraction efficiency, as reported in previous studies [59,60].

Previous studies with various types of propolis have also shown that ethanol is an important system modifier [35,61]. In a comparative study of extraction methods to obtain extracts from green propolis by SFE, Monroy et al. [36] found that when using 32% of the solution containing 80% ethanol and water as a cosolvent, a 43% increase in yield was obtained compared to SFE without the cosolvent, in addition to 220 mg GAE/g of phenolic compounds (extraction conditions 50 °C and 250 bar). Paviani et al. [53], when adding 15% ethanol as a cosolvent to obtain extracts of green propolis (extraction conditions 50 °C and 250 bar), obtained an increase of 43.7% compared to the same process without the use of a cosolvent. The influence of the addition and concentration of cosolvent on supercritical CO_2_ extraction was also shown for other natural matrices [62], demonstrating the importance of these modifiers to improve the extraction capacity of polar compounds. Cruz et al. [63] showed that the presence of hydrated ethanol (1/1, *v*/*v*) significantly increased the extraction yield of yacon leaves, obtaining extracts with higher antioxidant activity compared to extraction without the use of cosolvent, leading to higher yields. In a recent study, Guedes et al. [60] showed that as the concentration of ethanol added to the process was increased (0.5/1, 1.1/1 and 1.5/1, *w*/*w*), the yield and efficiency of SFE extraction in samples of *Synadenium grantii* increased.

**Figure 3:**
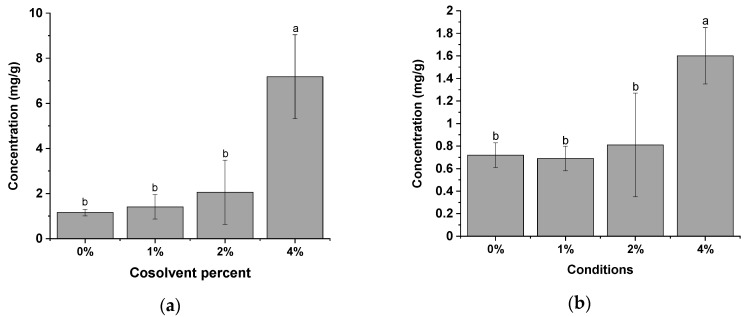
Determination of the levels of (**a**) Formononetin (mg/g); and (**b**) Kaempferol (mg/g) by HPLC-DAD in the extracts of red propolis obtained with the presence of different cosolvent percentages and using CO_2_ as a supercritical fluid (50 °C; 250 bar; CO_2_ flow rate of 6 g/min; S/F 131). Bars with the same letter are not significantly different (*p* < 0.05) according to Tukey’s test at the 95% confidence level.

The compound mainly found in the red propolis extract analyzed in this study was formononetin in the concentration range of 1 ± 0.2 mg/g (control 0%) to 7 ± 2 mg/g (extract with 4% of cosolvent), followed by kaempferol (from 1 ± 0.1 to 2 ± 0.3) (Figure 3). A 518% increase in the extraction capacity of formononetin was observed in the presence of 4% cosolvent in the system. For kaempferol an increase of 122% was observed.

Previous studies have also reported higher concentrations of phenolic compounds, such as formononetin, in extracts of red propolis, which is identified as its major component [4,15,22]. López et al. [64], when evaluating samples of red propolis from different regions by mass spectrometry, identified the presence of formononetin (*m*/*z* 267.06, retention time, rt 4.5 min) in all analyzed samples. Hanski et al. [65] demonstrated the antimicrobial effect of formononetin on *Chlamydia pneumoniae*, while Li et al. [66] demonstrated the protective effect of formononetin in vitro, decreasing the levels of TNF-α and IL-6. Thus, the pharmacological activities of red propolis may be closely related to their phenolic compounds due to their antioxidant, anti-inflammatory and microorganism proliferation inhibition capacity. Batista et al. [67] also associated the antioxidant and anti-inflammatory effects of red propolis with the involvement of polyphenols in the photoprotective activity observed in their study. These studies show the importance of obtaining extracts rich in formononetin for a greater biological capacity of the obtained product.

Despite the knowledge that the chemical composition of propolis depends on the biodiversity, type and geographical location of the beehives [22] and that the extraction method influences the extract composition [20,33], the identification of ideal conditions, especially regarding the use of clean technologies with the presence of adequate concentrations of system modifiers, given the polar nature of the compounds present in red propolis, is of great importance to obtain extracts with high antioxidant capacity, especially with high levels of formononetin. Formononetin has been associated with the antioxidant [68], antitumor [66], antimicrobial [22] and anti-inflammatory [8] effects of red propolis extracts. Lower contents of formononetin (6.15 and 6.54 mg/g) and kaempferol (0.43 and 0.65 mg/g) were identified by Reis et al. [23] in ethanolic extracts (ultrasound-assisted or not) of red propolis from the same geographical origin (Barra de Sto. Antonio, Porto Calvo, Alagoas, Brazil). Bueno-Silva et al. [69] evaluated the effect of season on chemical composition and found lower formononetin contents (78.76 to 112.78 μg/g) than those found in this study for ethanolic extract of red propolis of the same origin. This may demonstrate that SFE (CO_2_ as the solvent and ethanol as the cosolvent) may be an important and promising technology for obtaining red propolis extracts with potential for nutraceutical and cosmetic applications [70]. In the present study, it was possible to demonstrate that ethanol (as a cosolvent) played a role by causing an increase in the polarity and eluting power of supercritical CO_2_, maintaining the same process parameters without significantly changing selectivity.

### 2.3. Global Yield Isotherms (GYI)

**Figure 4:**
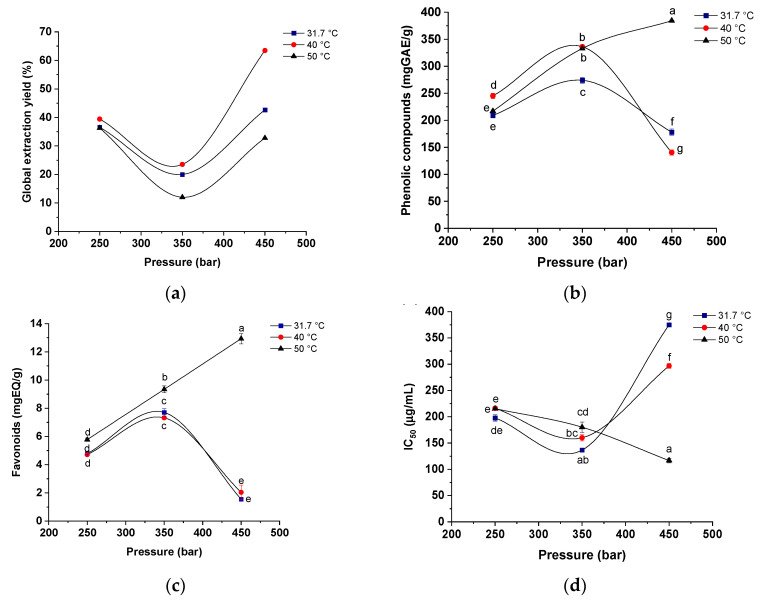
Global yield isotherms for (**a**) total yield percentage (%); (**b**) content of total phenolic compounds (mg EGA/g); (**c**) content of total flavonoids (mg EGA/g); and (**d**) antioxidant activity (IC50) (μg/mL) for the red propolis extracts using CO_2_ as supercritical fluid, ethanol as cosolvent (4% *w*/*w*) at temperatures of 31.7, 40 and 50 °C and pressures of 250, 350 and 450 bar (CO_2_ flow rate of 6 g/min and S/F of 131). Points with the same letter are not significantly different (*p* < 0.05) according to Tukey’s test at the 95% confidence level.

Figure 4 shows the global yield isotherms for total yield, total phenolic compound content, flavonoid content and antioxidant activity (IC_50_) determined for the red propolis extracts obtained under the different temperature (31.7, 40 and 50 °C) and pressure (250, 350 and 450 bar) conditions used (S/F 131-step 1; 4% ethanol-step 2).

Regarding yield, a higher extraction percentage (63.46%) was obtained at a pressure of 450 bar at 40 °C, while the highest content of phenolic compounds (380 mgEAG/g) and flavonoids (10 mg EQ/g) and higher antioxidant capacity (110 μg/mL) were obtained under 450 bar and 50 °C. Figure 4a shows that at constant pressures of 350 and 450 bar the increase in temperature significantly favors the extraction capacity, and consequently, the global yield of the process.

In general, the solubility of solutes in supercritical fluids increases with temperature at constant pressure [71]. When keeping the temperature constant at 31.7 and 40 °C, increasing the pressure from 350 to 450 bar also favors the process yield. The lowest yields at constant pressure were observed at the highest temperature (50 °C). It is known that the effect of temperature on SFE is complex due to the increase in the solute vapor pressure and reduction in the density of the supercritical solvent [72]. Increased temperature increases the vapor pressure of the solute, promoting an increase in its solubility in supercritical CO_2_; however, the temperature increase also promotes a reduction in the density of supercritical CO_2_, thus reducing the solubility of the solute in the solvent [73]. In this case, the effect of reduced supercritical CO_2_ density was favored in relation to the increased solvent density (Figure 4a).

In general, at a constant pressure of 250 bar, temperature had no effect on the extraction of phenolic compounds and total flavonoids or on the antioxidant activity (without significant differences). At the intermediate pressure studied (350 bar), variations in temperature promoted an increase in the extraction of these compounds in a sample of red propolis. For example, for phenols (40 and 50 °C), total flavonoids (50 °C) and antioxidant activity (50 °C), higher temperatures contributed to a higher extraction yield of these compounds. The positive effect of the temperature increase at a constant pressure of 450 bar (highest studied pressure) was clearly evident. Thus, the effect of temperature increase had a positive influence on the vapor pressure of the solutes [74] and, therefore, the temperature of 50 °C was the most efficient for obtaining extracts with high antioxidant capacity.

It is noteworthy that at constant pressure, the influence of temperature cannot be treated in such a simple way [75]. When evaluating the behavior of pressure in the 31.7 and 40 °C isotherms, it was observed that the increase in pressure from 250 to 350 bar contributes significantly to the increase in the extraction of phenolic compounds, flavonoids and antioxidant activity. However, a reduction in the levels of phenolic compounds and flavonoids, and consequently antioxidant capacity, was observed when the pressure increased from 350 to 450 bar in the 31.7 and 40 °C isotherms. Thus, at these temperatures, intermediate pressures (350 bar) may be more efficient for the extraction of phenolic compounds from red propolis.

The best phenolic and flavonoid yields, as well as antioxidant activity, were observed in the 50 °C isotherm at a pressure of 450 bar. In the 50 °C isotherm, the increase in pressure significantly accelerated the mass transfer in the supercritical extractor bed and contributed to increasing the extraction yield of phenolic compounds from red propolis. As demonstrated by Barroso et al. [76] and Fujii et al. [77], the density of supercritical CO_2_ is dependent on the pressure used in the extraction process; therefore, at higher pressures, supercritical CO_2_ will have a higher density, and consequently, its solvation power will be higher.

It is important to note that the isotherms crossed between 400 and 450 bar for total phenols (Figure 4b) and between 350 and 400 bar for antioxidant activity (Figure 4d). Below this pressure level, known as the crossover pressure, the solubility of the antioxidant compounds of red propolis decreases with increasing temperature. A crossover is observed near the critical region [78,79]; in these regions, the effect of temperature on the increase in vapor pressure compensates for the effect of temperature on decreasing solvent density [27]. Due to the existence of this point, the lowest and highest concentrations of phenolic compounds occurred in the same isotherm (50 °C). Azevedo et al. [80] evaluated the extraction of caffeine using SFE and found that near the critical point, below the crossover pressure, small increases in temperature result in a drastic decrease in the density of the solvent and, consequently, in its extraction capacity. For the extraction of artemisinin from *Artemisia annua* L., Rodrigues et al. [81] found a crossover pressure of 200 bar, whereas Cadena-Carrera et al. [82] evaluated the bioactive properties of the extracts from guayusa leaves (*Ilex guayusa* Loes.) and found a crossover pressure close to 200 bar.

**Figure 5:**
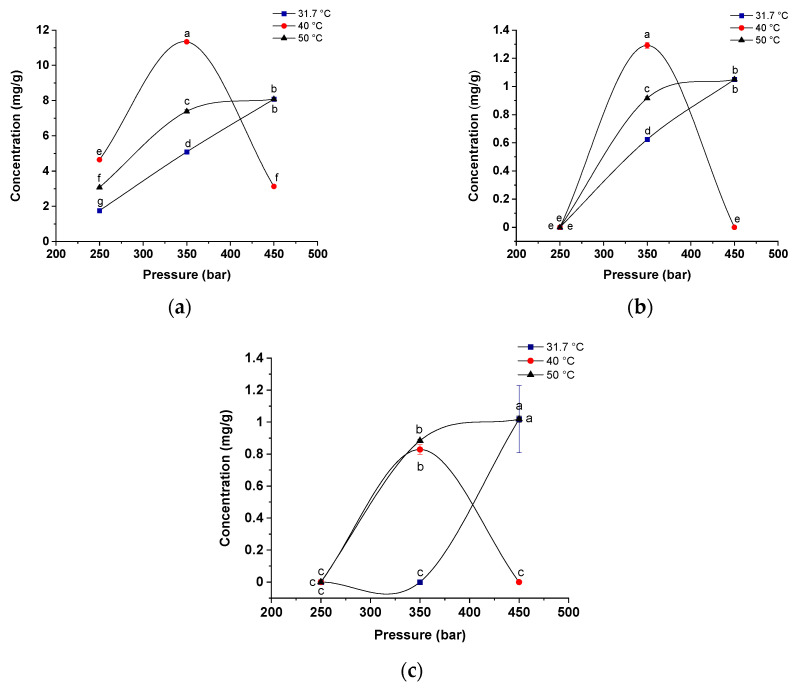
Isotherms for the concentration of: (**a**) formononetin; (**b**) naringenin; and (**c**) kaempferol for the extraction of red propolis using CO_2_ as the supercritical fluid, ethanol as the cosolvent (4%, *w*/*w*) at temperatures of 31.7, 40 and 50 °C and pressures of 250, 350 and 450 bar (CO_2_ flow rate of 6 g/min and S/F 131). Values that show the same letter in the same graph are not significantly different (*p* < 0.05) by the Tukey test at a confidence level of 95%.

**Figure 6:**
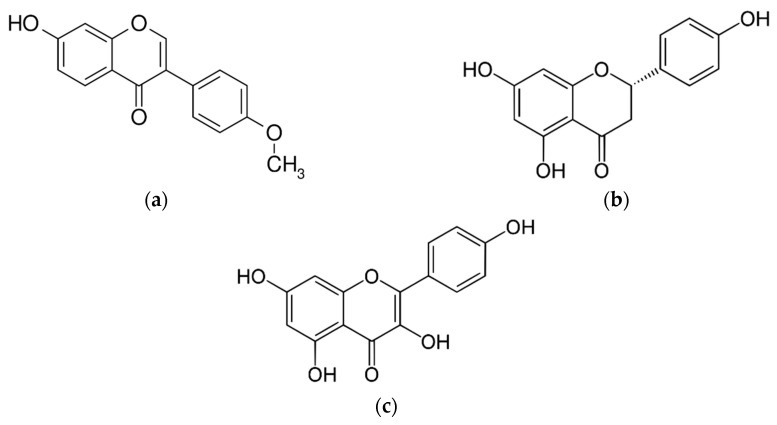
Chemical structures of phenolic compounds (**a**) Formononetin; (**b**) Narigenin; and (**c**) Kaempferol.

Figure 5 shows the global yield isotherms for the three compounds identified and quantified by HPLC-DAD ((a) formononetin; (b) naringenin; and (d) kaempferol)) in the red propolis extracts under the different temperature (31.7, 40 and 50 °C) and pressure (250, 350 and 450 bar) conditions employed (S/F 131-step 1; 4% ethanol-step 2) in this study. Figure 6 shows the chemical structure of each phenolic compound analyzed in this study.

Formononetin (Figure 5a) was obtained at higher concentrations (13 mg/g) at a pressure of 350 bar and a temperature of 40 °C (intermediate conditions). It is possible to observe that at constant pressures of 250 and 350 bar, there is an increase in the concentration of formononetin extracted when the temperature increases from 31.7 °C (2 mg/g and 6 mg/g) to 40 °C (5 mg/g and 10 mg/g), but there was a reduction at 50 °C (3 mg/g and 9 mg/g). In this case, the increase in temperature increased the vapor pressure of the solute, positively favoring the extraction process. However, at high temperatures, the effect of solvent density reduction may favor both the degradation of formononetin and the decrease in extraction efficiency for this compound [83,84]. In addition to solubility, the effect of temperature on the conversion or degradation of formononetin should also be taken into consideration. At a constant pressure of 450 bar, the highest concentration was observed at the highest and lowest temperatures employed, with concentrations of 9 mg/g and 7 mg/g, respectively. Thus, the effect of the supercritical CO_2_ solubility at 450 bar should be more strongly considered than the effect of conversion or degradation of formononetin in terms of extraction yield.

In addition, a crossover pressure close to 400 bar was also observed. When evaluating the pressure behavior at a constant temperature of 40 °C, an extraction profile similar to the extraction of total phenolic compounds, flavonoids and antioxidant activity was observed (Figure 5): when increasing the pressure from 250 to 350 bar, there is an increase in extraction, but when increasing the pressure from 350 to 450, extraction is reduced. Theoretically, the higher the pressure, the greater the density and solubility of the supercritical fluid, increasing the extraction efficiency [85]. However, the concentration of some of the target compounds may be reduced because at high pressures, other compounds can also be extracted, and thus, there is a reduction in specificity [86]. In general, high pressures may not be efficient for the extraction of formononetin because the effect of decreased diffusivity overlaps the effect of increased density, thus decreasing the extraction yield of this compound at 450 bar. A similar effect was found by Saito et al. [61] when obtaining phenolic compounds from green and red propolis, with higher yields (14%) observed at 60 °C and 200 bar and the lower yields (3.6%) under 40 °C and 300 bar.

Regarding the compound naringenin (Figure 5b), when analyzing the behavior of the isotherms at a constant pressure of 250 bar, it was observed that the temperature did not influence the extraction process. However, when the pressure increased to 350 bar, the effect of temperature is evident because by raising the temperature from 31.7 to 40 °C, an increase of 114% in extraction is obtained, and when raising the temperature from 40 to 50 °C, a reduction of 27.3% is observed. For this compound, crossing of the temperature curves was also observed at a pressure close to 400 bar, and a higher concentration (2 mg/g) at 40 °C and 350 bar. This behavior is in agreement with that observed for total phenolic compounds, flavonoids and antioxidant activity (Figure 5). When analyzing the behavior of pressure at constant temperature, it is noted that for the temperatures of 31.7 and 50 °C, the pressure has a positive effect on the extraction process. Under these conditions, the increase in pressure increases the density of the supercritical fluid and the solvation power of the solute, consequently increasing the extraction yield. At a temperature of 40 °C, extraction was only significant at a pressure of 350 bar. Majdoub et al. [87] found similar effects when raising the pressure from 100 to 300 bar at a constant temperature of 50 °C for obtaining extracts of *Daucus carota.*

Similar to what occurred for the compound naringenin, the temperature variation also did not influence the kaempferol extraction process (Figure 5c) at a constant pressure of 250 bar, and this compound may have its extraction reduced or become nonextractable under these conditions. However, at the pressure of 350 bar, there is an increase in extraction when the temperature increases from 31.7 to 40 and 50 °C (1 mg/g and 1.078 mg/g, respectively), but with no significant differences between the concentrations obtained at the higher temperatures. The isotherms cross at approximately 400 bar, and the influence of the crossover pressure on the extraction profile was evident because up to 400 bar pressure, the extraction is more effective at the lowest and highest temperatures studied, with no significant difference, and below this value, the extraction is more effective at 40 °C. Higher concentrations were obtained at a pressure of 450 bar and 50 °C (1 mg/g). Zordi et al. [51], in a study on SFE to obtain propolis extracts, also observed that the chemical composition of an extract is significantly influenced by pressure and temperature in both a linear and quadratic way, obtaining optimal operating conditions of highest yield (14.3%) in the isotherms of 317 bar and 45 °C.

Therefore, the present study shows that the conditions of global yield isotherms in SFE affect the composition of the extract obtained and should be analyzed and defined according to the extraction objective. In general, the best yields of bioactive compounds isolated from red propolis were observed at the intermediate isotherms of 40 °C and pressure of 350 bar.

## 3. Materials and Methods

### 3.1. Materials and Reagents

DMSO (dimethyl sulfoxide) and methanol were from Sigma-Aldrich Chemical Co., St. Louis, MO, USA, and acetic acid (standard HPLC) and ethyl alcohol (standard HPLC) were from EMSURE^®^, Merck, Darmstadt, Germany. A regenerated cellulose membrane filter, 0.45 μm (SLCR025NS, Millipore Co., Bedford, MA, USA) was used. Rutin hydrate (CAS number 207671-50-9), kaempferol (CAS number 520-18-3), formononetin (CAS number 485-72-3), gallic acid (CAS number 149-91-7), quercetin (CAS number 117-39-5), *p*-coumaric acid (CAS number 501-98-4), epicatechin (CAS number 490-46-0), caffeic acid (CAS number 331-39-5), catechin (CAS number 7295-85-4-4) and 2,2-diphenyl-1-picrylhydrazyl (DPPH●) (CAS number 1898-66-4) were purchased from Sigma-Aldrich Chemical Co. (St. Louis, MO, USA) and *trans*-ferulic acid (CAS number 537-98-4) was purchased from Fluka (St. Louis, MO, USA).

### 3.2. Study Sample

The sample of red propolis used in this study was kindly donated by the company Bee Product Natural (Barra de Sto. Antonio, Porto Calvo, Alagoas, Brazil) with “Mangroves of Alagoas” denomination of origin (IG201101) [88]. Approximately 1 kg of red propolis was ground (Cadence-Brazil) and sieved (270–325 μm diameter) to allow the homogenization of the sample in the extraction bed. Samples of 200 g of red propolis were kept at −30 °C in vacuum-sealed packaging away from light.

### 3.3. Process Parameters for Red Propolis Extraction by SFE

**Figure 7:**
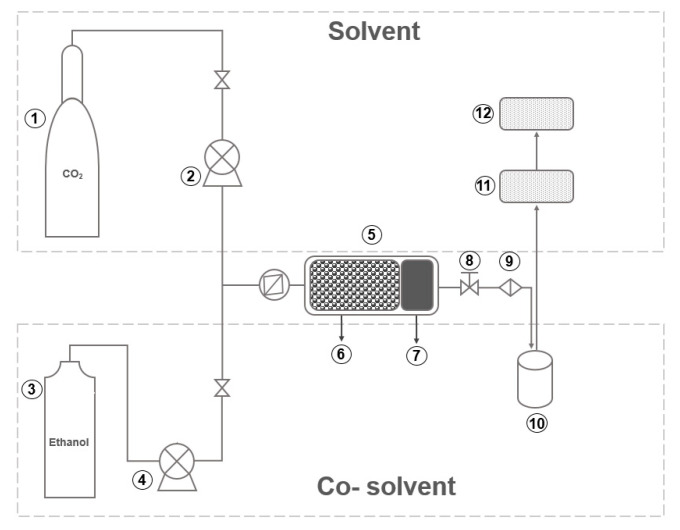
Schematic of the supercritical extraction system used in this study. 1—CO_2_ cylinder with dip tube; 2—CO_2_ pump; 3—Cosolvent cylinder; 4—Cosolvent pump; 5—Extraction cell; 6—Glass beads; 7—Sample (raw material); 8—Dynamic/static valve; 9—Restrictor valve; 10—Sample collection vial; 11—Flow meter; 12—Totalizer.

The process parameters used to obtain the extracts of red propolis was performed as previously described by Machado et al. [33] with modifications and using a SFT-110 Supercritical Fluid Extractor (Supercritical Fluid Technologies, Inc., Newark, NJ, USA) under the different conditions used in the study. The CO_2_ flow rate in the system was 6.0 g/min in all experiments (Figure 7).

**Figure 8:**
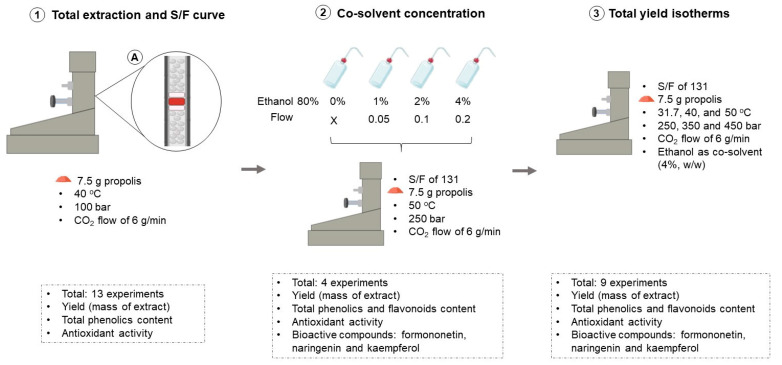
Illustrative summary of the steps, parameters used, total experiments and analyses performed at each step of the present study to obtain red propolis extract with high antioxidant capacity by SFE. **1**—First step: determination of the overall extraction curve and S/F (mass of CO_2_/mass of sample); **2**—Second step: influence of the cosolvent percentage; **3**—Third step: determination of the global yield isotherms. In A, the packing of the extraction bed to avoid the formation of preferential CO_2_ paths is shown.

The extraction bed was packed to avoid the formation of preferential paths by the solvent (CO_2_), and for this purpose, glass wool and beads were used to fully fill the bed (Figure 8). In this work, it 7.5 g of red propolis was used, as reported in previous optimization studies [33,53], mainly to avoid very long extraction times. The best extraction operational condition was determined in three steps. Figure 8 presents an illustrative summary of the process used with all parameters studied at each step, as well as the number of experiments and analyses performed. The results for the parameters evaluated and determined in this study were expressed as the mean ± standard deviation (*n* = 3), and all analyses were performed in triplicate.

#### 3.3.1. First Step: Overall Extraction Curve and S/F (Mass of CO_2_/Mass of Sample)

In the present study, to obtain the overall extraction curve, a temperature and pressure of 100 bar and 40 °C were used, respectively, with the objective of guaranteeing the worst extraction scenario (under mild extraction conditions), a sample of 7.5 g propolis and 6 g/min CO_2_ flow rate [33,49]. The experiment was performed as follows: the extracts were collected at predetermined periods in vials of previously known weight. The *S*/*F* value was calculated according to Equation (1).

(1) $$\frac{S}{F}=\frac{MCO_{2}}{M_{sample}}$$

where:

*MCO*_2_ = total mass (g) of CO_2_ used in the system at each extraction point (considering the volume and density of CO_2_ in the system)

*M_sample_* = total mass (g) of propolis used to feed the system

The overall yield (*X*_0_) was calculated in accordance with Equation (2).

(2) $$X_{0}=\frac{M_{extract}}{M_{sample}}$$

where:

*M_extract_* is the mass (g) of extract obtained in each extraction

*M_sample_* is the mass (g) of propolis used to compose the extraction bed

For the extracts obtained at the predetermined times (in each collection vial), the yield, total phenolic content and antioxidant activity were determined. A total of 13 experiments were obtained for each assay.

#### 3.3.2. Second Step: Influence of Cosolvent Percentage

In this step, 80% ethanol was used as a cosolvent to obtain the extracts in relation to the yield (in mass), content of total phenolic compounds, antioxidant activity and concentration of the compounds of interest (formononetin, naringenin and kaempferol). The extracts were obtained under the following conditions: S/F of 131 (calculated in the previous step), temperature of 50 °C and pressure of 250 bar (CO_2_ flow rate of 6 g/min) [33]. The extractions were performed using 0, 1, 2 and 4% of the cosolvent, calculated in relation to the mass of CO_2_ used (S/F), totaling 4 experiments for this step. Ethanol (80%) was diffused in the system using a cosolvent pump with a flow rate of 0.05 (1%), 0.1 (2%) and 0.2 (4%) mL/min for a total time of approximately 140 min of extraction (considering S/F = 131).

#### 3.3.3. Third Step: Global Yield Isotherms

The yield isotherms were studied using three temperatures (31.7, 40 and 50 °C) and three pressures (250, 350 and 450 bar). The *S*/*F* value used was obtained in the first step (131), and the percentage of cosolvent (80% ethanol) was determined in the previous step (4% *w*/*w*) (CO_2_ flow rate of 6 g/min). The extracts obtained under the different conditions were evaluated for yield (mass), content of total phenolics, flavonoids, antioxidant activity (EC_50_) and concentration of the compounds of interest (formononetin, naringenin and kaempferol). For this step, a total of 9 experiments were obtained for each test performed.

### 3.4. Total Phenolic Compounds, Flavonoids Content and Antioxidant Activity by DPPH● (2,2-Diphenyl-1-picrylhydrazyl)

The content of total phenolic compounds of the red propolis extracts was determined from the reaction with the Folin–Ciocalteu method [89,90]. The reaction was prepared as previously described by Devequi-Nunes et al. [20]. The results are expressed as milligram of gallic acid equivalent (GAE) per gram of sample (mg GAE/g). For this, a calibration curve (y = 0.0104x + 0.0688, R^2^ = 0.9976) was determined using standard solutions of the gallic acid (concentrations 0 and from 10 to 200 μg/mL).

The content of flavonoids of the extracts was determined using the method proposed by Meda et al. [91] with adaptations, as previously described by Machado et al. [21]. The same procedure was performed using standard solutions of quercetin (0 and from 1 to 75 μg/mL) to obtain a standard curve (y = 0.0311x + 0.0259, R^2^ = 0.9987). The content of total flavonoids was expressed as milligram of quercetin equivalent (QE) per gram of sample (mg QE/g).

To evaluate the antioxidant activity, the 2,2-diphenyl-1-picrylryrazine-reactive method (DPPH●) was applied [92,93], as previous described by Reis et al. [23]. The extracts were diluted to five concentrations (50, 100, 150, 200, 250 and 300 μg/mL) in triplicates. The free radical sequestration capacity was expressed as the percentage of radical oxidation inhibition (Equation (3)) (extracts obtained in the first and second study steps).

(3) $$AA\;\%=100-\left(\frac{Ab_{sample}\;\times 100}{Ab_{blank}}\right)$$

where:

*AA %* = antioxidant activity in percentage

*Ab_sample_* = absorbance of the extract sample

*Ab_blank_* = absorbance of the blank (without sample)

The IC_50_ value (effective concentration of the extract to sequester 50% of the DPPH● radical) was calculated based on the linear equation obtained from the extract concentrations and respective DPPH● radical sequestration percentages. For the extracts obtained in the third study step, the results are expressed as IC_50_.

### 3.5. High-Performance Liquid Chromatography (HPLC): Identification and Quantification of Phenolic Compounds

The red propolis extracts obtained by extraction with supercritical CO_2_ in the second (cosolvent influence) and third (yield isotherms) study steps were evaluated by high-performance liquid chromatography (HPLC—Shimadzu, LC-20AT, Japan equipped with an automatic injector and diode array detector—DAD, Shimadzu, SPD-M20, Kyoto, Japan) as previously described by Reis et al. [23], Salgueiro and Castro [94] and Cabral et al. [95]. For this, an analytical standard curve formed by 13 phenolic standards was obtained, and the presence of these compounds in the extracts was investigated. A NUCLEODUR 100-5 C18 ec column (150 × 4 mm internal diameter; 5 μm particle size) was used in conjunction with a ZORBAX Eclipse Plus C18 precolumn (4.6 × 12.5 mm) (Agilent, Folsom, CA, USA). The chromatographic analysis conditions were tested with an elution gradient with a mobile phase of 5% acetic acid (Phase A) and methanol (Phase B) at different proportions and with a total analysis time of 42 min (from 0 to 35 min [0–92% B]; 35 to 40 min [92–0% B]; 40 to 42 min [0% B]). The injection volume was 20 μL, and the flow rate was 1 mL.min^−1^. The device was operated at a temperature of 40 ± 2 °C. The DAD reading was adjusted in the range of 190 to 800 nm, and chromatographic acquisition was defined between 280 and 370 nm [23].

**Figure 9:**
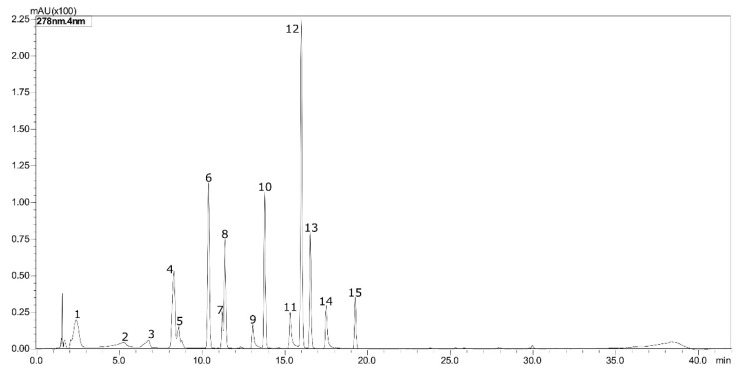
Chromatogram with the phenolic standards evaluated in this study by HPLC-DAD for the identification and quantification of the compounds present in the red propolis extracts obtained under different conditions (wave length = λ/retention time = RT/detection limit = DL/quantification limit = QL). 1—Gallic acid (RT 2.31 min, λ 280 nm, DL 0.47 mg/g, QL 1.58 mg/g); 2—*O*-dianiside (RT 4.92, λ 280, DL 0.62 mg/g, QL 2.50 mg/g); 3—Catechin (RT 6.51 min, λ 280 nm, DL 0.31 mg/g, QL 1.04 mg/g); 4—Caffeic acid (RT 8.15 min, λ 300 nm, DL 0.25 mg/g, QL 0.82 mg/g); 5—Epicatechin (RT 8.46 min, λ 280 nm, DL 0.20 mg/g, QL 0.68 mg/g); 6—*p*-coumaric acid (RT 10.32 min, λ 300 nm, DL 0.20 mg/g, QL 0.66 mg/g); 7—Rutin hydrate (RT 11.17 min, λ 320 nm, DL 0.35 mg/g, QL 1.15 mg/g); 8—*trans*-ferulic acid (RT 11.35 min, λ 320 nm, DL 0.26 mg/g, QL 0.86 mg/g); 9—Myricetin (RT 13 min, λ 370 nm, DL 0.30 mg/g, QL 1.00 mg/g); 10—Resveratrol (RT 13.77 min, λ 300 nm, DL 0.21 mg/g, QL 0.70 mg/g); 11—Quercetin (RT 15.36 min, λ 280 nm, DL 0.20 mg/g, QL 1.30 mg/g); 12—*trans*-Cinnamic (RT 15.99 min, λ 280 nm, DL 0.18 mg/g, QL 0.61 mg/g); 13—Naringenin (RT 16.53 min, λ 280 nm, DL 0.20 mg/g, QL 0.67 mg/g); 14—Kaempferol (RT 17.53 min, λ 320 nm, DL 0.24 mg/g, QL 0.82 mg/g); 15—Formononetin (RT 19.27 min, λ 300 nm, DL 0.19 mg/g, QL 0.64 mg/g).

The compounds were identified by comparing the retention time (RT) and the ultraviolet spectrum between samples and standards. The working range for all investigated compounds was 0.5 to 15 mg/g. The wave length (λ) ranged from 280 to 370. The RT ranged from 2.31 to 19.27 min, the detection limit (DL) ranged from 0.19 to 0.47 mg/g and the quantification limit (QL) ranged from 0.64 to 1.58 mg/g, and all these parameters were dependent on each compound. Figure 9 shows the chromatogram obtained for the construction of the analytical curve with the studied standards.

### 3.6. Statistical Analysis

The results were statistically analyzed using StatSoft 6.0 (StatSoft Inc., Tulsa, OK, USA). Analysis of variance (ANOVA) and Tukey’s test at the 95% confidence level were performed to identify significant differences between the results obtained for each test (*p* < 0.05).

## 4. Conclusions

This novel study showed the feasibility of applying SFE to obtain red propolis extracts with high antioxidant potential and that it is important to consider aspects related to process parameters, such as the volume of CO_2_ applied, addition of cosolvents and total yield isotherms. In the present study, the addition of ethanol as a cosolvent improved the extraction of bioactive compounds present in red propolis, including formononetin, the major compound of interest from this type of propolis. The best yield was obtained under the conditions of 40 °C and 450 bar using 4% ethanol as the cosolvent, a CO_2_ flow rate of 6 g/min and S/F of 131. The best conditions to obtain extracts rich in phenolic compounds, flavonoids and antioxidant activity (represented by a low IC_50_) were at a temperature of 50 °C and pressure of 450 bar. The intermediate conditions (40 °C and 350 bar) showed the greatest potential for obtaining high concentrations of formononetin and naringenin, as well as extracts with high antioxidant capacity. Formononetin is considered to be the compound with the greatest pharmacological interest in red propolis. In general, the presence of these compounds (formononetin, naringenin and kaempferol) at high concentrations in the extracts obtained in this study demonstrates, despite the low total yield, that SFE using CO_2_ is a promising alternative to obtain red propolis extracts with high added value. However, to obtain the specific bioactive compounds investigated in the present study, it is necessary to evaluate the individual properties of each one.

## Figures and Tables

**Table 1:** Determination of pilot extraction kinetics for red propolis using supercritical fluid extraction by CO_2_ with the results for S/F; mass yield of extract ± standard deviation (SD); mass yield of accumulated extract ± SD; total phenolic compounds in mg/GAE/g ± SD; and antioxidant activity (%) ± SD (parameters: 7.5 g of sample; 40 °C; 100 bar; CO_2_ flow rate 6.0 g/min).

Experiment Number	S/F	Mass Yield of Extract (g)	Accumulated Yield (%) and Extract Mass (g)	Phenolic Compounds (mg GAE/g)	Antioxidant Activity (%)
1	7.28	0.04 ± 0.01	1 (0.04 ± 0.01)	170 ± 20	20 ± 4
2	14.56	0.04 ± 0.01	1 (0.09 ± 0.01)	200 ± 10	30 ± 2
3	21.84	0.04 ± 0.01	2 (0.1 ± 0.01)	210 ± 4	20 ± 1
4	29.13	0.03 ± 0.01	2 (0.2 ± 0.01)	200 ± 20	20 ± 1
5	43.69	0.03 ± 0.01	3 (0.2 ± 0.01)	200 ± 10	30 ± 1
6	58.25	0.04 ± 0.01	3 (0.2 ± 0.01)	200 ± 20	30 ± 4
7	72.82	0.04 ± 0.01	4 (0.3 ± 0.01)	180 ± 20	30 ± 0.2
8	87.38	0.04 ± 0.01	4 (0.3 ± 0.01)	220 ± 10	30 ± 2
9	109.22	0.03 ± 0.01	4 (0.3 ± 0.01)	220 ± 5	30 ± 2
10	131.07	0.03 ± 0.01	5 (0.4 ± 0.01)	230 ± 21	30 ± 1
11	152.91	0.03 ± 0.01	5 (0.4 ± 0.01)	190 ± 20	20 ± 1
12	174.76	0.02 ± 0.01	6 (0.4 ± 0.01)	160 ± 1	20 ± 4
13	196.60	0.02 ± 0.01	6 (0.4 ± 0.01)	210 ± 30	20 ± 2

S/F = (mass of CO_2_/mass of sample).

**Table 2:** Results of the yields of total phenolic compounds (mg GAE/g), flavonoids (mg QE/g), IC_50_ (DPPH.) (μg/g), and the biomarkers formononetin (mg/g) and kaempferol (mg/g) using 1, 2 and 4% ethanol (cosolvent) in relation to the mass of CO_2_ (*w*/*w*) and without the presence of cosolvent (mean ± SD) (50 °C; 250 bar; CO_2_ flow rate of 6 g/min; S/F 131).

Analyses					
Cosolvent Parameters (% *w*/*w*)	Total Phenolic Compounds (mgGAE/g)	Flavonoids (mgQE/g)	IC_50_ (DPPH●) (µg/g)	Formononetin (mg/g)	Kaempferol (mg/g)
0	440 ± 40 ^a^	1 ± 0.03 ^c^	200 ± 2 ^b^	1 ± 0.2 ^b^	1 ± 0.1 ^b^
1	460 ± 70 ^a^	6 ± 0.20 ^b^	140 ± 2 ^a^	2 ± 1 ^b^	1 ± 1 ^b^
2	510 ± 80 ^a^	9 ± 0.1 ^a^	190 ± 12 ^b^	1 ± 1 ^b^	1 ± 0.1 ^b^
4	690 ± 200 ^a^	6 ± 2 ^b^	140 ± 14 ^a^	7 ± 2 ^a^	2 ± 0.3 ^a^

Values followed by the same letter in the column are not significantly different (*p* < 0.05) according to Tukey’s test at the 95% confidence level.
